# A Fast Parameter Identification Framework for Personalized Pharmacokinetics

**DOI:** 10.1038/s41598-019-50810-z

**Published:** 2019-10-02

**Authors:** Chenxi Yang, Negar Tavassolian, Wassim M. Haddad, James M. Bailey, Behnood Gholami

**Affiliations:** 1 Department of Electrical and Computer Engineering, Stevens Institute of Technology, Hoboken, NJ 07030 USA; 2 School of Aerospace Engineering, Georgia Institute of Technology, Atlanta, GA 30332 USA; 3 Northeast Georgia Medical Center, Gainesville, GA 30501 USA; 4 Autonomous Healthcare, Inc., Hoboken, NJ 07030 USA

**Keywords:** Data processing, Pharmacokinetics, Computational models

## Abstract

This paper introduces a novel framework for fast parameter identification of personalized pharmacokinetic problems. Given one sample observation of a new subject, the framework predicts the parameters of the subject based on prior knowledge from a pharmacokinetic database. The feasibility of this framework was demonstrated by developing a new algorithm based on the Cluster Newton method, namely the constrained Cluster Newton method, where the initial points of the parameters are constrained by the database. The algorithm was tested with the compartmental model of propofol on a database of 59 subjects. The average overall absolute percentage error based on constrained Cluster Newton method is 12.10% with the threshold approach, and 13.42% with the nearest-neighbor approach. The average computation time of one estimation is 13.10 seconds. Using parallel computing, the average computation time is reduced to 1.54 seconds, achieved with 12 parallel workers. The results suggest that the proposed framework can effectively improve the prediction accuracy of the pharmacokinetic parameters with limited observations in comparison to the conventional methods. Computation cost analyses indicate that the proposed framework can take advantage of parallel computing and provide solutions within practical response times, leading to fast and accurate parameter identification of pharmacokinetic problems.

## Introduction

Pharmacokinetics is defined as the study of the bodily absorption, distribution, excretion, and metabolism of a drug. Pharmacokinetics is mainly concerned with the effect of the body on the drug, in contrast to pharmacodynamics, which is mainly concerned with the effect of the drug on the body. These two disciplines comprise the field of clinical pharmacology^1^. The rational use of a drug requires an understanding of how the drug is absorbed and what the onset of action is, as well as an understanding of how long the drug will have an effect. The duration of the effect of a drug is determined by its *distribution* in various body tissue, its *metabolism*, and its *elimination* from the body. No drug can be approved by the United States Food and Drug Administration without the completion of a detailed pharmacokinetic analysis.

Most tissues (such as muscle or fat) are simply inert storage depots for the drug and do not chemically alter it; they rather serve to remove the drug from the circulation but do not eliminate it from the body. This process is called *distribution*. However, other tissues, most notably the liver and the kidneys, can chemically alter the drug, either to metabolites that still have some effect on the body, or to inactive metabolites. In addition, the kidneys can serve to eliminate the drug via urine. This process continues as blood cycles through the vascular bed. As the drug is eliminated by metabolic processes in the liver and kidneys, the drug that was stored in inert tissues by the distribution process leaves those storage depots and is recirculated undergoing metabolism and elimination from the body. Eventually, all drug stored in the body reaches the sites of metabolism and elimination.

From this abbreviated description, one can appreciate the potential complexity of the physiological processes that determine drug disposition. This complexity is in marked contrast to the simplicity of the data from a typical pharmacokinetic study. In a typical pharmacokinetic study, the investigator administers a fixed dose of the drug to a human volunteer or a patient with the disease the drug is expected to treat. Blood samples are then drawn at fixed intervals after the administration of the drug and the investigator determines the concentration of the drug in these samples. It is seldom possible to determine the drug concentration in the various tissues of the body. Thus, the investigator is left with essentially a table of drug concentrations in the blood as a function of time.

The ultimate goal of the investigator is to predict the drug concentration that would exist at times different from the sampling times of the study and for different doses, as well as to understand the variability that might exist between different subjects. Given the complexity of drug absorption, distribution, and elimination/metabolism and the sparseness of the data, it is inevitable that mathematical modeling plays a prominent role in pharmacokinetic analysis, bridging the gap between physiology and the available data. A major challenge in using pharmacokinetic models to predict patient response to a specific drug is the lack of knowledge regarding patient-specific parameters of the pharmacokinetic model^2,3^. One framework that is widely used in clinical practice to assist in the titration of different drugs is *population pharmacokinetics*, a statistical method to predict pharmacokinetic response based on a series of demographic and pathophysiological features of the patient.

Population pharmacokinetics has been covered under various topics, such as therapeutic drug monitoring, target concentration intervention, and Bayesian forecasting^4^. The Bayesian forecasting has been described extensively in the literature and has been implemented in practice for many drugs, especially antibiotics^5–8^. A number of softwares are also available for dose individualization^8,9^. The general frameworks behind these softwares are based on maximum likelihood estimation and mixed-effect modeling technique.

There are multiple factors contributing to the widespread adoption of the population pharmacokinetic framework. First, population pharmacokinetics provides an easy to use framework which does not involve complicated computations. In addition, calculating the appropriate drug dose involves the utilization of a statistical model and the corresponding statistical parameters. Once the raw pharmacokinetic data, generally collected during clinical studies, has been analyzed and statistical parameters of the population pharmacokinetic model are identified, access to raw data collected from previous patients is no longer needed^10^.

Although population pharmacokinetics has been used for decades, there are severe limitations associated with this approach. Specifically, population pharmacokinetics does not provide sufficient information to predict patient-specific pharmacokinetic response to drugs and only provides general pharmacokinetic behavior for a target patient population with limited prediction accuracy^11,12^. Additionally, certain assumptions regarding the statistical distribution of parameters (e.g., Gaussian), may not hold, which can affect the predictive performance of the model. Furthermore, the population pharmacokinetic approach is generally concerned with parameter identification of statistical models and fails to include validated mechanistic models which are based on the laws of physics (e.g., conservation of mass). Inclusion of mechanistic models for pharmacokinetics, which use ordinary differential equations (ODEs) that characterize drug distribution in the body, can provide a framework based on basic principles to characterize pharmacokinetic response and can increase prediction accuracy^13^. As the number of parameters to reproduce physiological functions tend to be large in pharmacokinetic models, efficient parameter estimation methods are essential.

Modern medicine is moving towards *personalized medicine*, where genetic analysis can guide personalized treatment^14^. Genetic analysis can also provide further insight on drug effect (i.e., pharmacodynamics) on individual patients. However, currently no framework exists for personalized pharmacokinetics to guide drug dosing. To ensure an effective personalized pharmacological treatment, we need to address interpatient and intrapatient variabilities in both pharmacokinetics and pharmacodynamics.

In this paper, we provide an approach to address the current gap in the continuum of pharmacological treatment by presenting a framework that can accurately predict pharmacokinetic response for a specific patient, which in turn can assist in personalized drug dosing. Specifically, a framework is presented that can use prior data as well as a limited number of measurements from the subject to accurately identify the parameters of a dynamical system model of drug pharmacokinetics for that subject. Such a dynamical system model with accurate parameter values can in turn be used to predict drug concentration over time, and hence, provide a framework to guide drug dosing. As discussed below, this problem involves complex computations, and hence, a numerical framework will be presented to solve the parameter identification problem in a reasonable time frame. The efficacy of the presented framework is established through analysis of pharmacokinetic data for the sedative drug propofol obtained from a dataset of 59 patients previously reported in the literature^15,16^.

## Compartmental Dynamical Systems

In this section, we provide a brief overview of the compartmental dynamical systems framework to model pharmacokinetics (see^17^ for a detailed discussion). Pharmacokinetic compartmental models make the assumption that the body consists of multiple compartments. Furthermore, the drug concentration is assumed to be uniform due to ideal mixing under each compartment. There are two major types of activities, i.e., the transportation to other compartments, and elimination from the body. These two activities are controlled by the metabolic processes of the human body. In the approximation of the real activities, the transport rate is usually assumed to be proportional to drug concentration. In addition, anotherapproximation is made that the mixing of the drug is instantaneous. This has marginal effect on the accuracy of the model as long as the estimation of drug concentration is not conducted right after the administration of the drug.

To formalize the parameter identification problem, we propose to model pharmacokinetics (PK) with compartmental dynamical systems. Specifically, drug concentration dynamics for a *n*-compartment system are given by the state space equation

1 $$\dot{x}(t)=f(x(t))+w(t),\,x(0)={x}_{0},\,t\ge 0,$$

where the drug concentration in each compartment is denoted by *x* = [*x*_1_, …, *x*_*n*_]^T^, the drug infusion from an external source is denoted by *w* = [*w*_1_, …, *w*_*n*_]^T^, and the mass exchange between different compartments are characterized by *f*(*x*) = [*f*_1_(*x*), …, *f*_*n*_(*x*)]^T^, where for *i* = 1, …, *n*,

2 $${f}_{i}(x)=-\,{\hat{a}}_{ii}(x)+\mathop{\sum }\limits_{j=1,\,i\ne j}^{n}\,[{\hat{a}}_{ij}(x)-{\hat{a}}_{ji}(x)],$$

with ${\hat{a}}_{ij}$ reflecting drug exchange coefficients between compartments.

The above model is general and can be used to model any pharmacokinetic system. For instance, a special case of the above nonlinear model is the *compartmental mammillary dynamical system*, which is often used to model the pharmacokinetics of certain drugs (e.g., the sedative propofol). Usually, a central compartment will be defined in the nonlinear mammillary model as the site for drug administration. The drug elimination through metabolism or other means occur from this central compartment. This compartment also connects to one or several peripheral compartments for the exchange of drug. The central compartment is considered to be a combination of blood within arteries and veins and the organs that have high blood flow to weight ratios. On the other side, the peripheral compartments are assumed to be metabolically inert as far as drug is concerned.

## The Parameter Identification Problem

In order to use a compartmental dynamical system to predict pharmacokinetic response, exact values of the parameters of the compartmental dynamical model (i.e., exchange coefficients between compartments, drug metabolism rate, etc.) are required. These values are unique to each individual patient and vary from patient to patient. An accurate knowledge of the compartmental model parameters can result in a more accurate prediction of the pharmacokinetic response of the patient for a given drug.

The parameter identification problem involves solving an *inverse problem*, where a set of measurements of drug concentration in the blood at multiple time points is given and the parameters of the compartmental dynamical system need to be identified^18^. This inverse problem is also referred to as *system identification* in the dynamical systems and controls literature. The inverse problem can be cast as an optimization problem, where a set of parameters need to be identified that minimize the error of model fit given the available data. The optimization problem associated with pharmacokinetics is generally *under-determined* (i.e., there are more unknowns than measurements), and hence, *ill-posed* (i.e., there are infinitely many solutions to the problem given the available information)^19^.

We denote unknown pharmacokinetic model parameters by $\theta \in {{\mathbb{R}}}^{n}$, where we assume that there are *n* unknown parameters. Furthermore, we assume that *m* noisy measurements *y*_1_, …, *y*_*m*_ at time instants *t*_1_, …, *t*_*m*_ are given, where *m* < *n*. The parameter identification problem involves solving the optimization problem

3 $${\rm{\min }}\,{J}_{y,\theta }(y,\theta ),$$

where *J*(*y*, *θ*) is a cost function quantifying model fit. Here, we define the cost function as

4 $$J(y,\theta )={\Vert y-F(\theta ,{t}_{1},\ldots ,{t}_{m})\Vert }_{2}^{2},$$

where *F*(*θ*, *t*_1_, …, *t*_*m*_) = [*F*_1_(*θ*, *t*_1_), …, *F*_*m*_(*θ*, *t*_*m*_)]^T^ denotes a mapping from model parameters *θ* to drug concentration at time instants *t*_1_, …, *t*_*m*_.

## Limitations of Using Current Optimization Methods for Solving the Pharmacokinetics Parameter Identification Problem

There are certain drawbacks associated with using existing optimization frameworks such as Levenberg-Marquardt (LM) for solving the parameter identification problem discussed in this paper as explained below.

### Long computation time

Each function evaluation in the optimization problem (3) (i.e., computing modeling error given a set of parameters) involves the solving a set of forward ordinary differential equations (ODEs) characterizing the compartmental pharmacokinetic model, which is computationally expensive. The optimization framework has to repeatedly solve the forward ODEs and update the set of parameters until the model error is minimized. The convergence of this process can be extremely slow. In addition, optimization methods such as the LM method are not appropriate for parallelization to reduce the computation time.

### Lack of an effective mechanism to use prior knowledge

As discussed above, current optimization frameworks allow one to choose an initial set of parameters *θ*_0_ for which the desired optimum needs to be sufficiently close. Current practice involves selecting an “average” set of values for this initial parameter set (which serves as our best estimate of parameter values). This is a drawback of current frameworks, as there is no “average patient” and “average values.” For personalized pharmacokinetics, we require an effective method for selecting the initial set of parameters such that they are as close as possible to the actual pharmacokinetic parameters.

## A Framework for Parameter Identification with Application to Personalized Pharmacokinetics

To address the limitations of current optimization methods, we present a framework known as the *Constrained Cluster Newton* (CCN) method to solve the parameter identification problem. This method has two main advantages as compared to more traditional methods for solving the inverse problem. First, it provides a mechanism to include pharmacokinetic data collected from previous patients to guide the optimization process. Second, it involves dividing the problem into a series of independent sub-problems that will allow parallel computing to reduce computation time.

The proposed Constrained Cluster Newton method is an extension to the Cluster Newton method^3^. The Cluster Newton method is used to find a family of solutions for an under-determined inverse problem in a way that is significantly more efficient than solving a series of independent optimization problems with different initialization values (see^3^). The Cluster Newton method starts with a *cluster* of initial points, which are generally sampled from a region defined by average pharmacokinetic values and ranges reported in the literature. It then forward-solves the ODEs for each point in the cluster. Next, it fits a hyperplane to the solutions using a least squares framework to obtain a linear approximation of the function characterizing the forward problem (which is usually nonlinear). All the initial points are collectively moved closer to the solution via an update step. By repeating the above steps, the cluster of points will move close to the solution(s) of the inverse problem. The accuracy of the estimated solutions can then be further improved using *Broyden’s* method until the desired accuracy is achieved^3,20^.

In an extension to the Cluster Newton method (CN), which we call the Constrained Cluster Newton method, we can use data from prior patients to guide the optimization process by imposing constraints on the initial cluster of points. Specifically, we assume that a dataset including blood concentrations overtime is available for a specific drug. Next, instead of randomly sampling points uniformly from a relatively large region defined by the range of values determined through population pharmacokinetics, we identify a set of patients in the archived dataset that have the most *similar* pharmacokinetic response to the patient under consideration to generate a more *targeted* cluster of points, used for initialization of the optimization framework.

**Figure 1:**
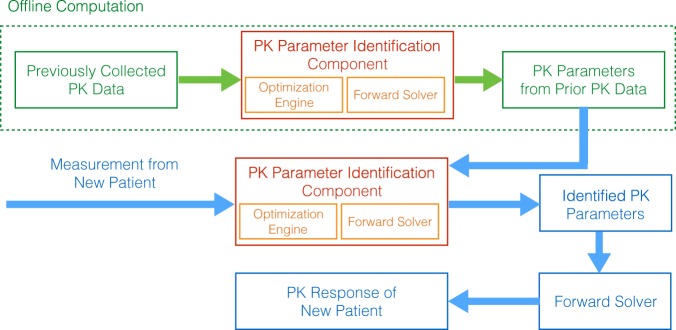
The overall architecture of the fast personalized pharmacokinetics framework.

The overall architecture of the fast personalized pharmacokinetics framework is shown in Fig. 1. Initially, the parameter identification component is performed offline (and once) to compute the pharmacokinetic parameters for previously collected data from a set of patients (i.e., drug concentration over time). Next, when the pharmacokinetic response of a new patient is needed, the framework identifies the most *similar* patients in the archived dataset to complement the drug concentration measurement obtained from the new patient. Finally, once the pharmacokinetic parameters for the new patient are identified, they are used in conjunction with a forward-solver to compute the pharmacokinetic response of the patient to the drug (e.g., the area under the drug concentration over time curve, maximum concentration, half life, etc.). Details of these processes are discussed below.

### Identifying the parameters of patients in the archived dataset

First, an inverse problem for each patient in the archived dataset is solved such that one set of pharmacokinetic parameters is obtained for each patient. This process is performed once and offline. Specifically, if there are *q* patients in the archived dataset, then we identify pharmacokinetic parameters *θ*_*j*_, *j* = 1, …, *q*, for the *j*th patient by minimizing *J*(*y*_*j*_, *θ*), where $J(\,\cdot \,,\,\cdot \,)$ is defined by (4) and *y*_*j*_ = [*y*_*j*,1_, …, *y*_*j*,*mq*_]^T^ is the set of measurements available for the *j*th patient in the archived dataset. Here, we use the LM method; however, other frameworks may be used.

### Initial estimate of the parameters for the new patient

Next, we solve the inverse problem (3) for the new patient under consideration to identify an initial estimate of the parameters $\tilde{\theta }$. This is performed by solving a standard parameter identification problem by initializing the optimization parameters using average pharmacokinetic parameter values determined from the archived dataset or published in the literature. While the identified parameter $\tilde{\theta }$ using this step is not accurate, it serves as our best estimate of the approximate region in the parameter space where the actual parameters reside.

### Using prior data from the archived dataset to predict pharmacokinetic response

Finally, we select a set of patients from the archived dataset with a pharmacokinetic response that is closest to the new patient under consideration. Patients with the most similar pharmacokinetics can be selected either by selecting any patient from the archived dataset with his/her pharmacokinetic parameters being within a specific distance from the new patient (threshold-based), or by selecting the *n* patients from the archived dataset who are closest to the new patient (*n*-nearest neighbor). In the threshold-based approach, a patient from the archived dataset is considered similar to the current patient if $\parallel \theta -\tilde{\theta }\parallel \le \alpha$, where *θ* is the pharmacokinetic parameters of the patient selected from the archived dataset, $\tilde{\theta }$ is the initial estimate for the new patient, and *α* > 0 is a specified threshold. Alternatively, if the *n*-nearest neighbor is used, then *n* patients with the smallest distance $\parallel \theta -\tilde{\theta }\parallel$ are selected.

**Figure 2:**
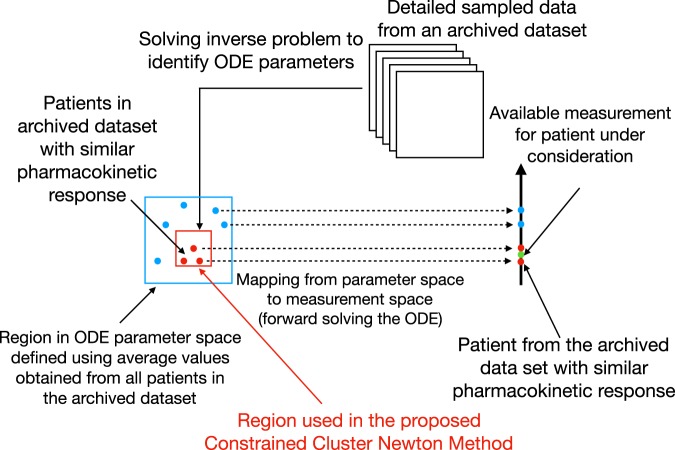
A schematic of the proposed Constrained Cluster Newton method.

Once the set of patients from the archived dataset is selected, their identified pharmacokinetic parameters are used to define a *targeted* region and generate a cluster of points to use with the Cluster Newton method. Note that the choice of the cluster used for initializing the optimization process has a significant impact on the results. Hence, selecting *similar* patients from the archived dataset can improve the results by defining a more targeted region to use for generating the initial cluster. Here, we choose a minimum bounding box to define the region from which the cluster is randomly sampled. Once the pharmacokinetic parameters of the new patient are identified, the pharmacokinetic response of the patient to a dose of drug can be computed by forward solving the ODEs using the identified parameters. A schematic of the proposed approach outlined in Sections 2 to 2 is shown in Fig. 2.

## Application to Personalized Pharmacokinetics for the Sedative Drug Propofol

In this section, we model the pharmacokinetics of the sedative agent propofol^21^ as an example for the proposed framework. Propofol is a fast-acting intravenous hypnotic agent. It can produce *anxiolysis* when being used in low doses. In higher doses, it causes *hypnosis*. Propofol may be administered as a bolus or continuously. Propofol depresses the myocardial contractility, which leads to decreased cardiac output. Furthermore, the decrease in cardiac output affects the redistribution kinetics, which is related to the blood transportation from the central compartment to the peripheral compartments. As a consequence, the drug concentration in the central compartment may be increased, leading to more depression in myocardial contractility, and oversedation.

**Figure 3:**
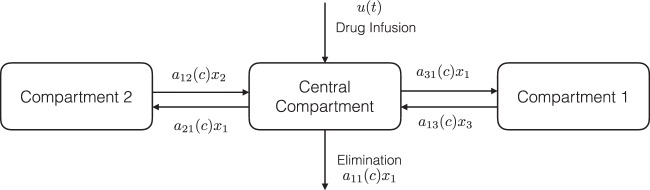
The three-compartment model of propofol.

The pharmacokinetics of propofol are described by the three-compartment model^17,22^ shown in Fig. 3. As shown in Fig. 3, *x*_1_ represents the mass of drug in the central compartment. The remaining portion of the drug in the body is assumed to reside in two peripheral compartments. The masses are shown as *x*_2_ and *x*_3_, respectively. Specifically, *x*_2_ is associated with muscle and the *x*_3_ is related with fat.

A three-compartments dynamical model is defined as below

5 $$\begin{array}{rcl}{\dot{x}}_{1}(t) & = & -[{a}_{11}(c(t))+{a}_{21}(c(t))+{a}_{31}(c(t))]{x}_{1}(t)\\  &  & +{a}_{12}(c(t)){x}_{2}(t)+{a}_{13}(c(t)){x}_{3}(t)+u(t),\,{x}_{1}(0)={x}_{10},\,t\ge 0,\\ {\dot{x}}_{2}(t) & = & {a}_{21}(c(t)){x}_{1}(t)-{a}_{12}(c(t)){x}_{2}(t),\,{x}_{2}(0)={x}_{20},\\ {\dot{x}}_{3}(t) & = & {a}_{31}(c(t)){x}_{1}(t)-{a}_{13}(c(t)){x}_{3}(t),\,{x}_{3}(0)={x}_{30},\end{array}$$

where $c(t)=\frac{{x}_{1}(t)}{{V}_{c}}$, *V*_c_ is the volume of the central compartment (about 15 $\ell$ for a 70-kg patient), *a*_*ij*_(*c*), *i* ≠ *j*, is the rate of transfer of drug from the *j*th to the *i*th compartment, *a*_11_(*c*) is the rate of drug metabolism and elimination (metabolism typically occurs in the liver), and *u*(*t*), *t* > 0, is the infusion rate of the sedative drug propofol into the central compartment. The transfer coefficients are assumed to be functions of the drug concentration *c* since it is well known that the pharmacokinetics of propofol are influenced by cardiac output^23^ and, in turn, cardiac output is influenced by propofol plasma concentrations, both due to *venodilation*^24^ and myocardial depression^25^.

Experimental data indicate that the transfer coefficients *a*_*ij*_(⋅) are nonincreasing functions of the propofol concentration^24,25^.

The most widely used empirical models for pharmacodynamic concentration-effect relationships are modifications of the Hill equation^26^. Applying this almost ubiquitous empirical model to the relationship between transfer coefficients implies that

6 $${a}_{ij}(c)={A}_{ij}{Q}_{ij}(c),\,{Q}_{ij}(c)=\frac{{Q}_{0}{\tilde{C}}_{50,ij}^{{\alpha }_{ij}}}{{\tilde{C}}_{50,ij}^{{\alpha }_{ij}}+{c}^{{\alpha }_{ij}}},$$

where, for *i*, *j* ∈ {1, 2, 3}, *i* ≠ *j*, ${\tilde{C}}_{50,ij}$ is the drug concentration associated with a 50% decrease in the transfer coefficient, *α*_*ij*_ is a parameter that determines the steepness of the concentration-effect relationship, and *A*_*ij*_ are positive constants. It is to be noted that both pharmacokinetic parameters are functions of *i* and *j*, that is, there are distinct Hill equations for each transfer coefficient. Furthermore, since for many drugs the rate of metabolism *a*_11_(*c*) is proportional to the rate of transport of drug to the liver, we assume that *a*_11_(*c*) is also proportional to the cardiac output so that *a*_11_(*c*) = *A*_11_*Q*_11_(*c*).

## Evaluating the Performance of the Personalized Pharmacokinetic Framework for Dosing of Propofol

In this section, we apply the presented framework to predict the pharmacokinetic response of a patient to propofol.

### Identifying the parameters of patients in the archived dataset

First, we use data reported in^15^ and^16^, where plasma concentrations from arterial samples were collected from 59 adult volunteers (age 23–82 and weight 57–114 kg). Subjects received a propofol infusion of 0.5 mcg/kg/min for an average time of 8 minutes. Times at which blood sampling was performed varied across subjects. However, approximately 17 samples were collected from *t* = 0 to *t* = 100 min from all subjects. In addition, depending on the subject’s condition, between 0 to 10 extra samples were collected from subjects after *t* = 100 min.

As a first step, pharmacokinetic parameters for subjects in the archived dataset were identified using the Levenberg-Marquardt method. Furthermore, since there were variations in total infused propofol among different subjects in the archived dataset, the pharmacokinetic response of each subject in the archived dataset was updated using the drug dose of the new patient.

### Using Prior data from the archived dataset to predict pharmacokinetic response

We compare the prediction accuracy of three frameworks: *i*) the Levenberg-Marquardt (LM) method, where no prior data is available and only typical ranges of pharmacokinetic parameter values are provided; *ii*) the Cluster Newton (CN) method, where no prior data is available and only typical ranges of pharmacokinetic parameter values are provided; and *iii*) the Constrained Cluster Newton (CCN) method, where data from prior patients are available.

In order to initialize the LM and CN methods, a cluster of 500 points is generated to be used by the optimization algorithms, where the cluster is chosen from the range given by typical values of the pharmacokinetic parameters reported in the literature^22,27^. The parameters are assumed to be distributed in a normal distribution with the mean values centered as *A*_11_*Q*_0_ = 0.119, *A*_21_*Q*_0_ = 0.112, *A*_12_*Q*_0_ = 0.055, *A*_31_*Q*_0_ = 0.0419, *A*_13_*Q*_0_ = 0.0033, and *V*_c_ = 15.96. The standard deviation of these values are std(*A*_11_*Q*_0_) = 0.0351, std(*A*_21_*Q*_0_) = 0.1051, std(*A*_12_*Q*_0_) = 0.0458, std(*A*_31_*Q*_0_) = 0.0155, std(*A*_13_*Q*_0_) = 0.0013, and std(*V*_c_) = 12.7. In contrast, in the CCN method, the archived dataset is used to generate the cluster of 500 points to initiate the optimization process as discussed in Section 1. In addition, we use both the threshold-based approach (with the threshold set to *α* = 5) and the nearest neighbor-based approach (with *n* set to 20) to solve the parameter identification problem, namely CCN(th) and CCN(nn), respectively.

### Evaluation of the proposed method

We use a *leave-one-subject-out* method, where data from 58 patients are assigned to the archived dataset and the remaining one patient is considered as a new patient. This process was repeated 59 times so that each subject in the dataset was considered as the new patient and all the others were assigned to the archived dataset. In the end, all results were aggregated. To solve the personalized pharmacokinetic problem, we assumed that drug concentration in plasma for the new patient is only available at one time instant (at approximately *t* = 3 min depending on the availability of data in the dataset). Our goal was to compare the prediction accuracy of the personalized pharmacokinetic approach with the ground truth established by the available data for each subject not included in the analysis.

In both CN and CCN methods, the optimization was performed for 10 iterations. However, Broyden’s method was not used at the end of each iteration. We used the LM implementation in the MATLAB optimization toolbox (version 2018)^28^. In the LM method, the optimization was terminated when the last step was smaller than the function tolerance of 10^−6^ or when the number of iterations was larger than 400^29^. To compare the pharmacokinetic prediction accuracy of each method, the following performance metrics were used: (*i*) peak concentration of the drug in the first 100 minutes denoted by *C*_*max*_, (*ii*) time to maximum concentration *C*_*max*_ denoted by *t*_*max*_, (*iii*) half-life defined as time to half *C*_*max*_ denoted by *t*_*half*_, and (*iv*) area under the curve (AUC) obtained by integration of the concentration-time curve. Here, we consider the area of a single-dose curve from time 0 to 100 minutes given by

7 $$AU{C}_{0-100}={\int }_{0}^{100}\,y(t){\rm{d}}t,$$

where *y*(*t*) represents the concentration-time relationship.

All metrics are reported as absolute percentage errors (APE) by comparing the estimated values to the available ground truth. The performance metrics for LM and CN methods were compared to the proposed CCN methods (both the threshold-based and the nearest neighbor approaches).

**Figure 4:**
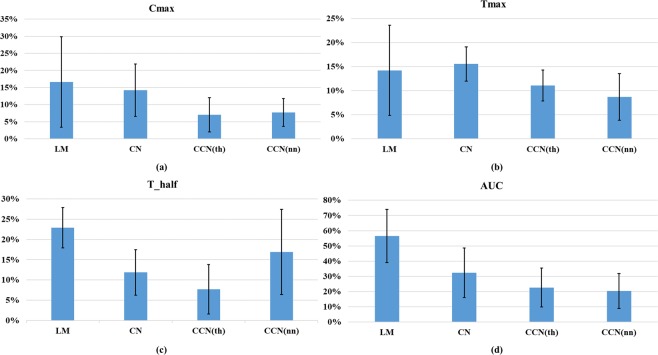
Comparison of the leave-out validation results in (**a**) absolute percentage error (APE) of the maximum concentration *C*_*max*_; (**b**) APE of the time to the maximum concentration *t*_*max*_; (**c**) APE of the half-life *t*_*half*_; (**d**) APE of the area under the curve *AUC*_0−100_. (Bar: average, Error Bar: standard deviation).

Figure 4 illustrates the error values of the results from all methods. The values in Fig. 4(a–d) are presented in the average APE ± standard deviation. It can be seen that the errors from the CCN method are smaller than the values from the CN and LM methods for most of the metrics regardless of the specific subject selection method used in CCN (nearest neighbor or threshold-based). In addition, the conventional CN method shows better results in average APE in *C*_*max*_, *t*_*half*_, and *AUC*_0−100_ compared to the conventional LM method. This result matches the observation in^3^ and can be explained by the fact that the CN method uses multiple sets of initial points, whereas the LM method solves the inverse problem with only one set of initial points. This improves the accuracy of the estimation from CN method.

Detailed results are given in Table 1. The threshold-based CCN, that is, CCN(th), gives a better performance in comparison to nearest-neighbor-based CCN method, that is, CCN(nn), regarding *C*_*max*_ (7.03% in comparison to 7.71%) and *t*_*half*_ (7.69% compared to 16.89%). However, CCN(nn) shows better performances compared to CCN(th) in *t*_*max*_ and *AUC*_0–100_, reporting 8.67% versus 11.06% and 20.39% versus 22.62%, respectively. Taking the average of the four APEs, CCN(th) shows a better value of 12.10% ± 6.79% compared to 13.42% ± 7.72% from CCN(nn).

**Table 1:** Performance Metrics for Different Pharmacokinetic Parameter Identification Frameworks.

Method	*C*_*max*_		*t*_*max*_		*t*_*half*_		*AUC*_0−100_	
	Mean	SD	Mean	SD	Mean	SD	Mean	SD
LM	16.62%	13.22%	14.20%	9.36%	22.91%	5.00%	56.54%	17.51%
CN	14.23%	7.68%	15.53%	3.54%	11.88%	5.62%	32.31%	16.21%
CCN(th)	7.03%	5.03%	11.06%	3.20%	7.69%	6.10%	22.62%	12.81%
CCN(nn)	7.71%	4.04%	8.67%	4.84%	16.89%	10.53%	20.39%	11.48%

In summary, the proposed CCN method for personalized pharmacokinetics provides a more accurate prediction of pharmacokinetic response based on the performance metrics defined above. Although CCN(th) shows better overall performance in terms of the average APE of the four metrics in Table 1, it is superior to CCN(nn) only in two out of the four metrics. Therefore, we can deduce that the performances of both CCN(th) and CCN(nn) are comparable.

## Reducing Computation Time through Parallel Computing

In order to utilize a personalized pharmacokinetic framework in a clinical setting, minimal computation time is critical. One limitation of using traditional methods such as Levenberg-Marquardt (LM) is that the computational structure is not well-suited for a parallel computing framework, leading to long computation times of hours and days. One attractive property of the newly-developed Constrained Cluster Newton method compared to LM is that it possesses the appropriate architecture for running the algorithm on multiple processors. Specifically, the process involves solving a number of independent forward solutions to ODEs, and then, updating the parameters. Each independent forward solution corresponds to a point randomly sampled from the initialization region. Running the program on multiple CPUs significantly reduces the computation time as discussed below.

To address the computational inefficiency of the numerical method, the *computational bottleneck* needs to be identified. Our analysis showed that more than 90% of the total computation time is dedicated to solve the ODEs. However, the CCN method involves independent solution to multiple ODEs, and hence, they can be solved in parallel. We used a workstation with 2 Intel Xeon CPU E5-2650 at 2.00 GHz (8 core 16 threads) running MATLAB to solve the parameter identification method using CCN. The workstation allows MATLAB to use a maximum of 12 workers in a parallel pool. A worker, defined under the MATLAB environment, is a processing thread that can be used for parallel calculation tasks. The MATLAB program manages workers by aggregating them into a pool. The workers in the pool can be used interactively and communicate with each other during the parameter identification task. The major parallel computation load is the ‘parfor’ function of the code, which is used to forward-solve the ODEs in the pharmacokinetic model.

**Figure 5:**
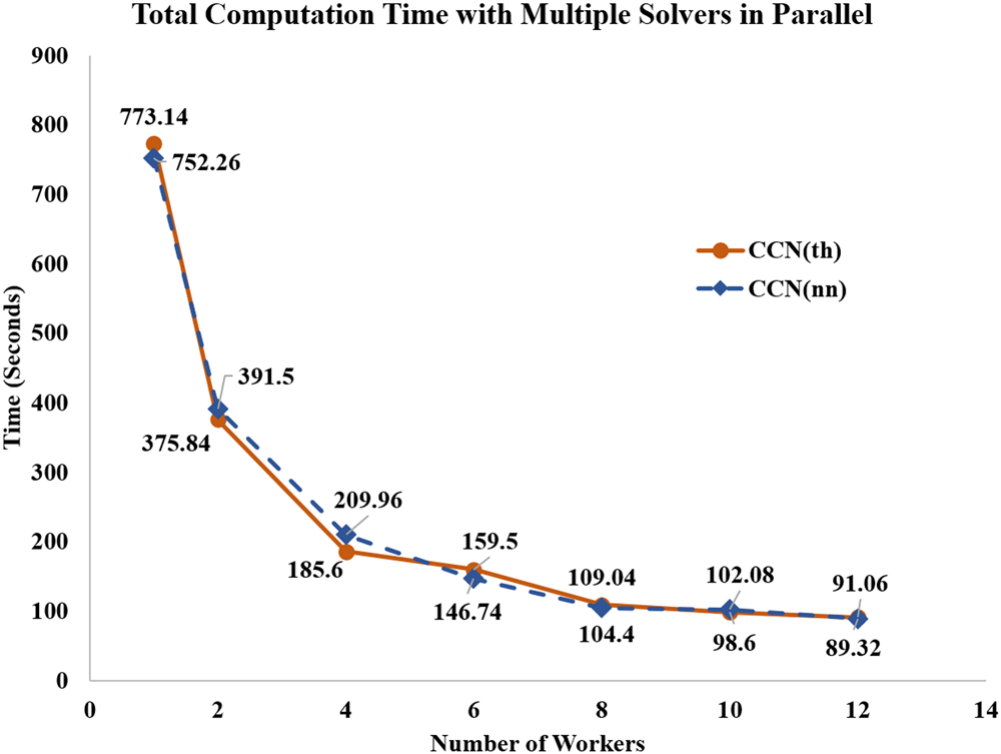
Comparison of the computation time with different number of workers.

During the test, both the CCN(nn) and the CCN(th) methods are executed with 59 subjects and 10 iterations for each observation (590 iterations in total). The same test is evaluated with 1, 2, 4, 6, 8, 10, and 12 workers. The results are shown in Fig. 5. The total computation time from the CCN(th) method with one worker is 773.14 seconds. The average computation time of one subject is 13.10 seconds. The time significantly drops to 375.84 seconds in total and 6.37 seconds on average when we have two workers working in parallel. The benefit from increasing the number of workers decreases slightly as we change from 2 workers to 4, 6, 8, 10, and 12 workers with reported total times of 185.61, 159.50, 109.04, 98.60, 91.06 seconds, respectively. Furthermore, the average computation time of the estimation from one subject with 4, 6, 8, 10, and 12 workers is 3.15, 2.69, 1.85, 1.67, and 1.54 seconds.

Alternatively, the total computation time from the CCN(nn) method with one worker is 752.26 seconds with an average of 12.75 seconds per subject. The computation time decreases to 391.51 seconds and the average computation time is reduced to 6.64 seconds when 2 workers are used. The computation time continues to drop as the number of workers increases. As illustrated in Fig. 5, the total computation time is 209.96, 146.74, 104.41, 102.08, and 89.32 seconds with 4, 6, 8, 10, and 12 workers, respectively. On average, the computation time for one subject is 3.56 seconds with 4 workers, 2.49 seconds with 6 workers, 1.77 seconds with 8 workers, 1.73 seconds with 10 workers, and 1.51 seconds with 12 workers.

It can be observed that both the CCN(nn) and CCN(th) methods received significant benefits when the worker numbers are increased from one to two. Both methods also reveal a similar trend of receiving less significant improvements from the increased number of workers when more than two workers are involved. For instance, the average computation time from CCN(nn) with 1 worker is 13.10 seconds. This value is reduced to 1.54 seconds with 12 workers, with an improvement ratio of 8.50 (13.10/1.54). However, this ratio is lower than the worker ratio of 12 (12/1). By segmenting the computation time into detailed algorithm components, we observed that the scheduling and distribution of the parallel calculation tasks are the major reason that the benefit from parallel computing becomes less significant for a larger number of workers. The ratio of worker management time to total computation time is higher for a larger number of workers. Finally, it was also observed that the computation time of the LM method is much higher than the CCN method with the same initial points, reporting more than 7400 seconds for the LM method versus 13.10 seconds from the CCN(nn) method.

## Discussion and Conclusion

In this paper, we presented a framework for fast parameter identification for personalized pharmacokinetic applications. Our numerical experiments validate the feasibility of this framework to improve the accuracy of pharmacokinetic parameter estimations with limited observations. The best overall performance is achieved with the constrained Cluster Newton method (CCN) employing a threshold strategy. Experimental results also indicate that it is beneficial to apply parallel computing to the CCN method.

In future research, we will evaluate the sensitivity of the algorithm to dosage variations and improve the robustness of the algorithm. Extensive tests with other pharmacokinetic models will also be conducted to evaluate the model-dependency of the algorithm. One limitation of this study is that the database is relatively small. In our future studies, we will evaluate the performance of the algorithm with a larger database. Another limitation is that our framework requires a high number of samplings in the *archived dataset*. This will ensure that the dynamics of the new patient can be adequately characterized by the proposed least-squares-based approach. In our future studies, we will investigate the effects of an increase or a decrease in the sampling density on the performance of the framework.

To achieve fast computation, more workers are needed. Implementation of the proposed framework on Graphic Processing Units (GPU) could be beneficial in this regard, as ODE implementation is reported to be 65 times faster on GPU than on CPU^30^. This will reduce the response time to the level of milli-seconds per estimation task, pushing forward this research to practical applications.

## Data Availability

The datasets generated during and/or analysed during the current study are available in the OPENTCI repository, http://opentci.org/data/propofol.
